# The Intricate Puzzle of Adrenocortical Tumors: Revisitation of Two Old Cases of Virilizing Adrenocortical Neoplasia with Contradictory Diagnostic and Histopathological Findings and Opposite Conclusions

**DOI:** 10.3390/life15121902

**Published:** 2025-12-12

**Authors:** Antonio Bellastella, Silvia Mercadante

**Affiliations:** Department of Cardiothoracic and Respiratory Sciences, University of Campania “Luigi Vanvitelli”, 80131 Naples, Italy; silvia.mercadante@studenti.unicampania.it

**Keywords:** adrenal tumors, diagnosis, therapy, prognosis

## Abstract

Two old cases of adrenocortical tumors with contradictory diagnostic findings and opposite conclusions are revisited in light of the recent WHO guidelines, as follows: a 34-year-old woman and a 15-month-old girl, both with severe virilization and adrenal mass at radiological investigation, studied in 1966 and 1977, respectively, in accordance with the diagnostic procedures available in those years. Dynamic hormonal findings seemed to exclude malignancy in the woman but were in favor of malignancy in the girl. Instead, a 305 gr mass on the right adrenal gland was removed in the woman and histopathologically verified as adrenocortical carcinoma, whereas in the girl, a 140 gr mass in the right adrenal gland was removed and histopathologically verified as adrenocortical adenoma. After a six-month span of clinical condition improvement, the woman developed recurrence with multi-organ metastases. Mitotane treatment temporarily improved her condition, but it progressively worsened until her death 11 months later. The girl instead showed progressive improvement in clinical and laboratory findings until complete normalization in 18 months. The use of dated radiological and laboratory investigations suggests caution against generalization of our assumption; however, these cases suggest that only histopathological findings from surgical specimens ensure a correct diagnosis of adrenocortical masses.

## 1. Introduction

Rapid female virilization may be linked to adrenocortical pathologies. These include diffuse adrenal cortical hyperplasia, adrenocortical nodular disease, adrenocortical adenomas and, particularly, adrenocortical carcinomas. This carcinoma is a rare endocrine malignancy with an estimated incidence of 0.7–2 cases per million/year, often with an unfavorable prognosis. Endocrine, neoplastic, and molecular aspects of adrenocortical proliferation have been systematically classified by the new World Health Organization (WHO) guidelines [1]. Two old cases of adrenocortical tumors with contradictory diagnostic findings and different conclusions are revisited in the current article, as follows: a 34-year-old woman and a 15-month-old girl, both with severe virilization, studied in 1966 [2] and 1977 [3], respectively, in accordance with the diagnostic procedures available in those years. We also aim to retrospectively verify whether the application of the current guidelines could have optimized diagnosis, therapy, and prognosis of these two patients.

## 2. Case 1

**Figure 1:**
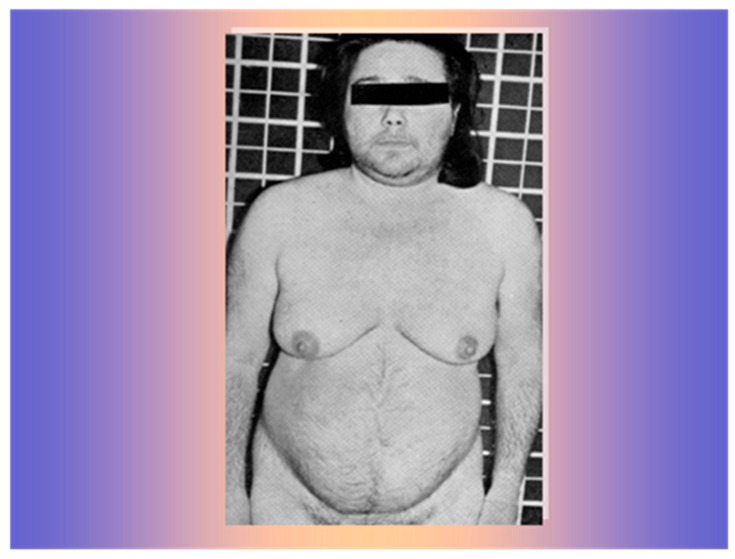
The 34-year-old women with frontal hair loss and widespread increase in heterosexual hair growth.

A 34-year-old woman was referred at the Medical Semeiotics Units of University of Naples in February 1966. She was married and had had four full-term pregnancies without complications. After her last birth in 1964, she had noticed progressive menstrual disorders until the occurrence of amenorrhea, hair loss, widespread increase in heterosexual hair growth, muscle hypertrophy, and severe arterial hypertension (Figure 1).

**Figure 2:**
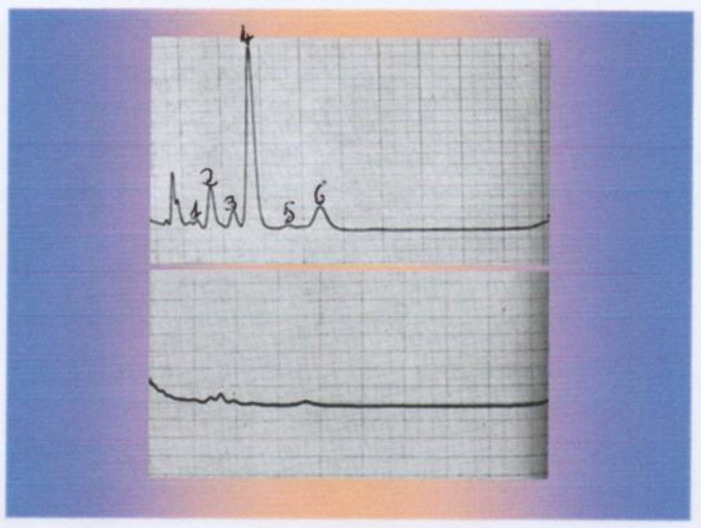
Gas-chromatographic determination of the 24 h urinary 17-Ketosteroids before (**upper** graph) and after dexamethasone inhibition (3 mg/day for 5 days: **lower** graph). 1: Pregnanediol, 2: Androsterone, 3: Etiocolanolone, 4: Dehydroepiandrosterone, 5: Pregnanetriol, 6: 11-Oxo Androsterone.

Radiological findings showed a large mass on the superior margin of the right adrenal gland (image not available). Laboratory findings showed normal plasma cortisol and adrenocorticotropic hormone (ACTH) levels but increased levels of urinary androgens, especially dehydroepiandrosterone (DHEA), evidenced by the gas-chromatographic determination of the 24 h urinary 17-ketosteroids but with significant inhibition after dexamethasone (3 mg/day for 5 days: Figure 2), an unusual finding in adrenal malignancy [4].

**Figure 3:**
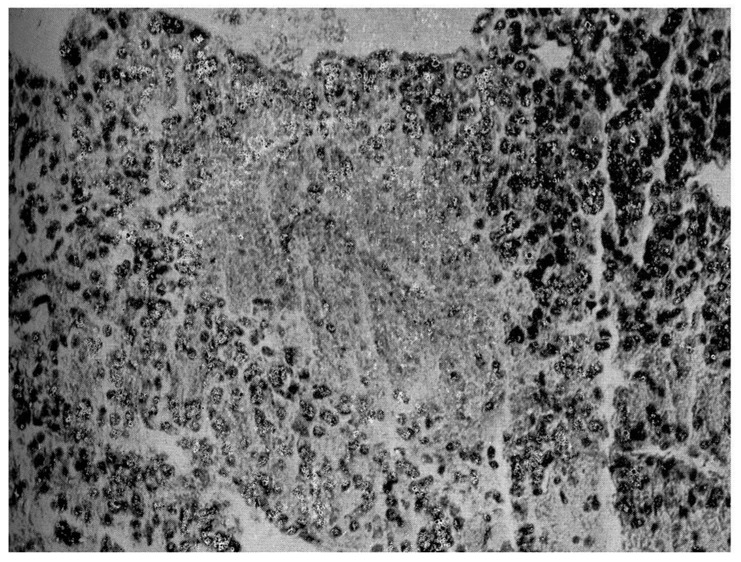
Histopathological findings on surgical specimen: widespread subversion of glandular architecture with high nuclear grade, high mitotic grade, atypical mitoses, and vascular and capsule invasion supporting the diagnosis of adrenocortical carcinoma, also along the lines indicated by the more recent WHO overview [1,5].

Instead, when transferred to the Surgical Semeiotics Unit for surgery, the surgeon, during the laparotomy, found a large 305 gr mass attached to the superior pole of the adrenal gland, which was removed and histopathologically verified as adrenocortical carcinoma (Figure 3).

After the surgical treatment, the patient showed a progressive improvement in clinical condition with the recovery of menstrual cycle, normal blood pressure, acne, and hair loss over six months. Then, she slowly showed a recurrence of hypertension, acne, hair growth, and amenorrhea associated with a new increase in urinary androgen excretion. A laparoscopic exploration showed two masses located above the right kidney and posterior to the inferior vena cava, respectively, which were removed and histopathologically found to be metastases of adrenocortical carcinoma. The patient started adjuvant therapy with O,p-DDD (mitotane), in those years a new tested anticancer drug, kindly provided to us by the Department of Health National Cancer Institute, USA. The initial dose was 2 gr/die until progressively reaching a dose of 10 gr/die in 15 days. In the first days of this treatment, the patient experienced transient nausea and hyperchlorhydria, which was controlled with antiacid therapy. A new progressive improvement in clinical and laboratory findings was observed, which persisted until the tenth month of treatment, without significant liver, kidney, and blood alterations. Then, suddenly showing hair loss and amenorrhea again, she was hospitalized again. Tests revealed a new increase in urinary androgen excretion. The patient suddenly suffered from violent nausea and uncontrollable vomiting associated with profound asthenia. The physical examination revealed the presence of a layer of fluid in the abdomen and severe hypotension. The situation rapidly worsened with circulatory collapse from which she did not recover, which led to her death despite the timely start of intensive therapy with hydrocortisone and adrenaline.

## 3. Case 2

**Figure 4:**
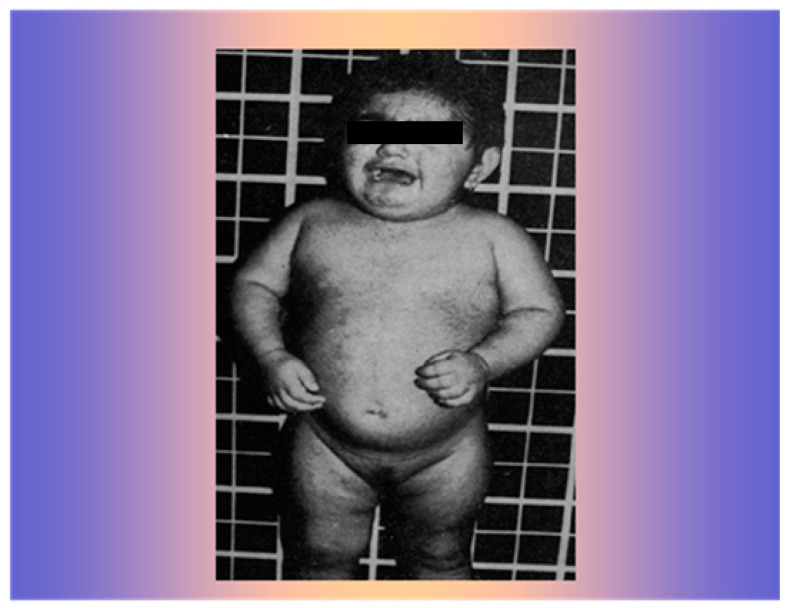
The 15-month-old girl with widespread increase in iso- and heterosexual hair growth and muscle hypertrophy.

A 15-month-old girl was referred at the Consitutional Medicine Unit of the University of Naples in October 1977. Since birth, she had shown an increase in isosexual and heterosexual hair, as well as muscle and clitoris hypertrophy (Figure 4).

**Figure 5:**
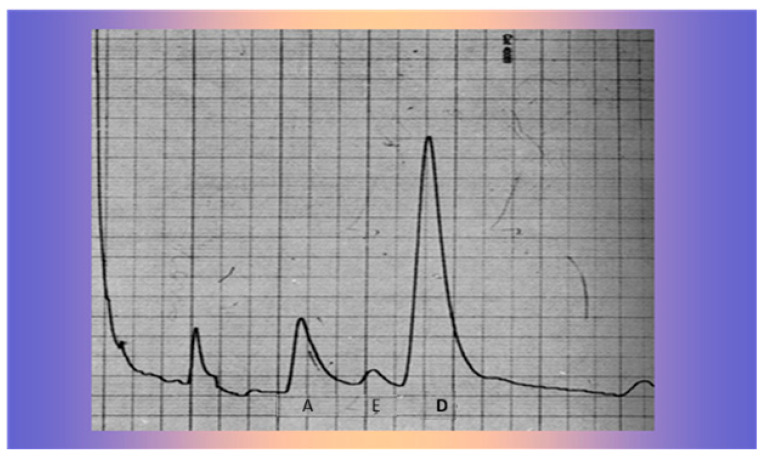
Gas-chromatographic determination of 24 h urinary 17-Ketosteroids A: Androsterone, E: Etiocolanolone, D: Dehydroepiandrosterone.

She showed blood pressure 90/55, a weight and height age of approximately 20 months, and a bone age of 38 months by the Tanner–Witehouse 2 (TW2) method. Laboratory findings showed normal plasma ACTH and cortisol levels but increased levels of urinary androgens, especially DHEA (Figure 5), as evidenced by gas-chromatographic determination of the 24 h urinary 17-ketosteroids, without significant inhibition after dexamethasone.

**Figure 6:**
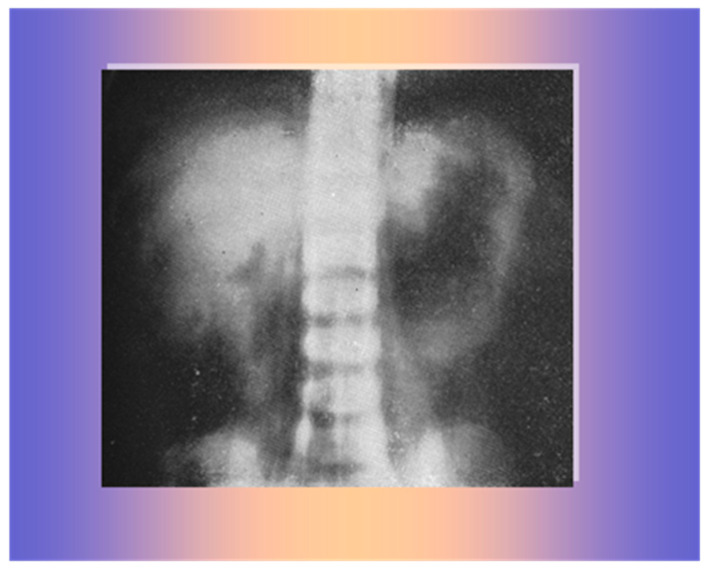
A large mass on the superior margin of the right kidney.

An abdominal X-ray showed a large mass likely of adrenal origin above the right kidney (Figure 6).

**Figure 7:**
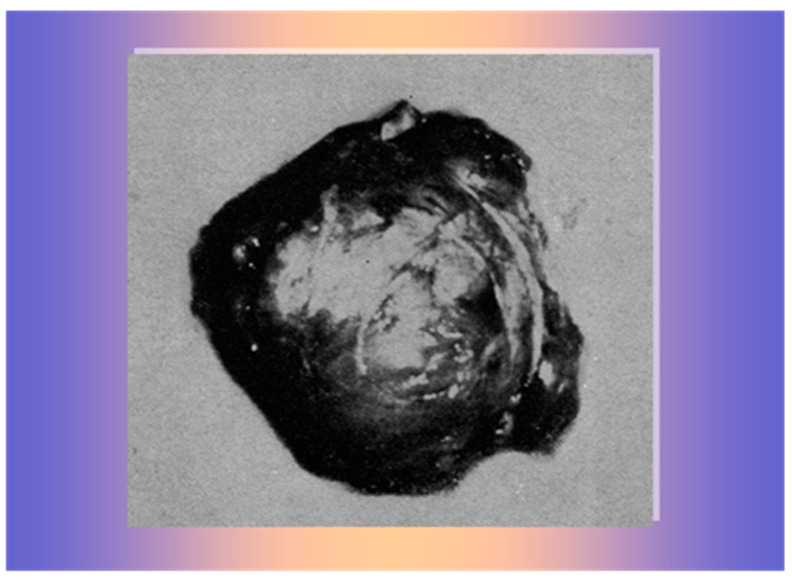
A 140 gr brownish, encapsulated mass, histologically verified as adrenocortical adenoma.

Then, she was transferred to the Surgical Unit for surgery for suspected adrenal carcinoma. During the laparotomy, the surgeon found and removed a large, brownish, encapsulated mass above the right kidney, adjacent to the inferior vena cava (Figure 7), histopathologically verified as adrenocortical adenoma without any sign of cellular atypia. The left adrenal gland appeared normal.

**Figure 8:**
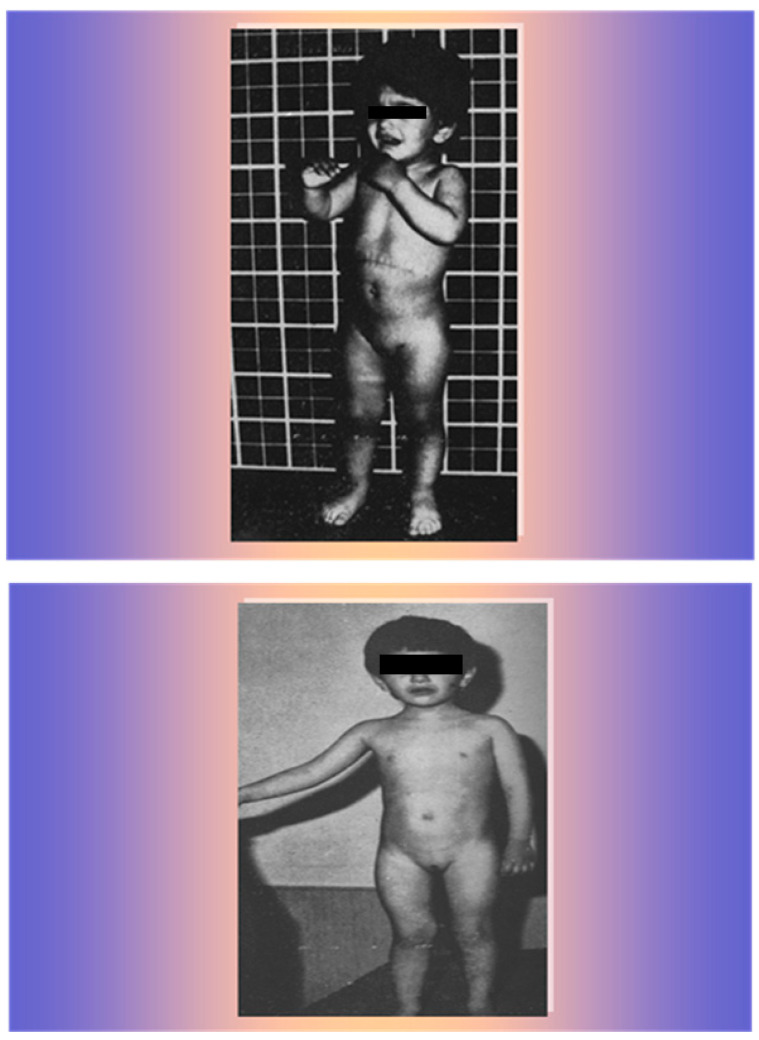
The patient 9 months after surgery (**upper** photo) and 18 months after surgery (**lower** photo).

The patient showed a rapid improvement in clinical and hormonal findings with progressive acne regression, hair loss, and muscle and clitoris normalization, gaining weight, height, and a normal appearance for her sex and age within 18 months (Figure 8).

## 4. Discussion

Hyperandrogenism in women typically presents clinically with hirsutism, acne, muscle hypertrophy, and/or androgenic alopecia. The underlying etiology may be different in pediatric and adult ages. In adult women, the most frequent disorder causing androgen excess is polycystic ovary syndrome (PCOS), which affects up to 10% of all women. Other hormonal virilizing alterations include congenital adrenal hyperplasia, Cushing’s disease, and androgen-secreting tumors of the ovary or adrenal gland. These disorders may be missed in the absence of an appropriate screening approach [6]. Detailed clinical history, physical examination, and hormonal phenotyping may help detect women who are at the highest risk of non-PCOS disorders. Rapid-onset symptoms, overt virilization, postmenopausal onset, or severe hormonal disturbances should prompt investigations for underlying neoplastic pathology, including dynamic testing and imaging, where appropriate [6,7,8,9]. In particular, searching for virilizing tumors with morphological and functional evaluation, not available at the time of our two studies, is now mandatory [7]. In particular, morphological (by computed tomography and Hounsfield units) and functional (by fluorodeoxyglucose positron emission tomography) evaluation may help diagnose secreting tumors, including adrenal ones [8,9]. In pediatric age, adrenocortical tumors are rare, representing ~0.2% of all pediatric malignancies and having an incidence of 0.2–0.3 new cases per million per year in the United States [7]. At diagnosis, most children show signs and symptoms of virilization, but some show a discrepancy between clinical and hormonal findings, with scarce clinical alterations in spite of abnormal concentrations of adrenocortical hormones and genetic and/or epigenetic alterations, represented by germline TP53 mutations or chromosome 11p abnormalities [7]. Recently, the new WHO guidelines have systematically classified endocrine, neoplastic, and molecular aspects of adrenocortical proliferations [1,5]. Along the lines indicated by these guidelines and in light of their criteria, we decided to revisit these two old cases of adrenocortical tumors for their apparently confounding clinical, laboratory, imaging, and even surgical diagnostic findings, and for their opposite conclusions. The current study was also aimed at retrospectively verifying whether the application of these guidelines could have optimized diagnosis, therapy, and prognosis of these two patients. In the first case, clinical findings of a widespread increase in heterosexual hair growth, muscle hypertrophy, amenorrhea, and severe arterial hypertension oriented the diagnosis towards an adrenal cancer. Radiological and basal hormonal findings, in particular, the large mass on the superior margin of the right adrenal gland and high levels of urinary androgens excretion, especially DHEA, seemed to support this diagnosis. However, the inhibition of this excretion close to zero under dexamethasone test, an unusual finding in adrenal malignancy [4], seemed to fuel the hope of a benign process, even if this behavior has rarely been observed in cases of malignant masses [4]. Instead, surgical and histopathological findings confirmed the diagnosis of adrenocortical carcinoma. The mitotane is now considered a possible therapeutic choice for patients with recurrence of adrenocortical carcinoma, even if this drug is recommended as adjuvant therapy in patients at standard or high risk of recurrence but not in those with low-grade, localized, adrenocortical carcinoma [10]. In those years, this was a new tested anticancer drug, kindly provided to us by the Department of Health National Cancer Institute, USA. Our patient already met the most recent criteria for indicating this adjuvant treatment, since she had had metastatic dissemination. In fact, her condition improved significantly and remained stable for ten months until a sudden, rapid worsening that led to her death in short time, in spite of timely appropriate therapy. In the second case, the presence since birth of clinical findings, including isosexual and heterosexual hair and muscle and clitoris hypertrophy, seemed to orient the diagnosis towards an adrenal hypersecretory cancer, even if these clinical alterations may have been caused by other more benign adrenal pathologies [7]. This seemed to be supported by hormonal findings of increased excretion of urinary androgens, especially DHEA, without inhibition after dexamethasone test, even if this behavior may also be observed in some cases of benign masses. Moreover, the surgical findings with the removal of a 140 gr mass seemed to confirm malignancy. In fact, usually, an adrenal mass being malignant has been shown to increase significantly with size (greatest diameter): a malignant/benign ratio of 8:1 has been reported for masses over 4 cm in diameter and over 100 gr in weight [4,7,9], all characteristics observed in our case. Instead, fortunately, the histopathological findings and the natural history of our patient, in good health after surgery and in the following years up to today, confirmed the diagnosis of adrenal adenoma, one of the major lesson(s) gained from those historical cases.

## 5. Conclusions

Obviously, our study has important limitations related to the use of dated radiological and laboratory investigations that suggest caution against the generalization of our assumptions; however, we believe that it may be of some interest for its implications for clinical practice. Moreover, our results also show that the application of the current guidelines could have not significantly improved diagnosis, therapy, and prognosis of our two patients. In conclusion, we believe that the revisitation of these two old cases indicates that in patients affected by adrenal masses, clinical, laboratory, and imaging findings can be controversial and sometimes contradictory, and only the histopathological findings from surgical specimens ensure a correct diagnosis of these masses. Considering this, an interesting lesson emerging from these historical cases is that large adrenal masses (over 10 cm in diameter) should all be taken seriously and removed via an open approach to avoid dissemination by pneumoperitoneum established during laparoscopic or robotic procedure [11,12]. In conclusion, the revisitation of these two cases adds a small piece to the intricate puzzle of adrenocortical tumors, whose correct and early diagnosis, as well as the choice of which tailored therapy, still constitutes a challenge for physicians in clinical practice.

## Data Availability

Further data regarding these two cases are unavailable due privacy and ethical restrictions.
